# Total elbow arthroplasty as a reconstruction option for distal humerus osteosarcoma: A case report^☆^

**DOI:** 10.1016/j.ijscr.2021.106392

**Published:** 2021-09-08

**Authors:** Uno Surgery Erwin, Sigit Daru Cahyadi

**Affiliations:** a Department of Orthopaedic and Traumatology, Faculty of Medicine Universitas Indonesia, Dr. Cipto Mangunkusumo Hospital, Indonesia; b Department of Orthopaedic and Traumatology, Faculty of Medicine Universitas Indonesia, Persahabatan General Hospital, Indonesia

**Keywords:** Osteosarcoma, Distal humerus, Total elbow arthroplasty

## Abstract

**Introduction:**

Osteosarcoma is the second most common malignant bone tumor. The humerus is the third most common anatomical location for osteosarcoma, however, osteosarcoma around the elbow joint is uncommon. The intricacy of the elbow joint, limited soft tissue coverage, and proximity to nerves and arteries make the surgical resection and reconstruction complicated.

**Case report:**

A 17-year-old boy came with a chief complaint of lump and pain on his left elbow. One month later, the patient felt there was a lump with a size of a marble on the left elbow, which getting bigger to a size of a tenis ball. Physical examination showed mass on the posterior aspect of the elbow with the size 14x12cm. Plain radiographs revealed osteolytic lesion and periosteal reaction of the distal humerus and the magnetic resonance imaging (MRI) showed a low intensity on T2-weighted imaging. Histopathological examination suggested osteosarcoma. The patient underwent neoadjuvant chemotherapy for 3 cycles. The patient was treated with limb salvage surgery by wide excision, cryosurgery followed by total elbow arthroplasty and ORIF with plate and screw. Postoperative plain radiographs showed the plate and screws are well-fixated. The patient can slowly regain his elbow motion without limitation one month postoperatively.

**Conclusions:**

Distal humerus in an unusual site for osteosarcoma. Total elbow arthroplasty and ORIF with plate and screw is a favorable reconstruction option for distal humerus osteosarcoma with excellent postoperative functional outcomes.

## Highlights

• Osteosarcoma around elbow joint is very rare
• Limb salvage surgery is the standard procedure for the osteosarcoma, but the involvement of elbow joint made the decision for preservation difficult
• Total elbow arthroplasty and ORIF with plate and screw for distal humerus osteosarcoma provides good functional outcome

## 1 Introduction

Osteosarcoma is the second most common malignant tumor of the bone after multiple myeloma. Osteosarcoma is common in adolescents, with 75% of all the cases developed between 15 and 25 years of age [1]. The anatomical predilection of osteosarcoma includes the metaphyseal area of the femur, tibia, humerus, and pelvis. The humerus is the third most common anatomical location to develop osteosarcoma, with 10% of all osteosarcoma cases [2]. However, the development of osteosarcoma around the elbow joint is uncommon, with an incidence of less than 1% [3]. Current treatment for osteosarcoma includes chemotherapy, radiotherapy, and complete surgical resection [1]. Nowadays, limb salvage surgery is more common than amputation with the advantage of improved functional outcomes after surgery [2]. However, wide surgical excision with adequate surgical margin should be achieved to suppress local recurrence.

Due to limited data, the intricacy of the elbow joint, limited soft tissue coverage, and proximity to nerves and arteries, surgical resection, and reconstruction in distal humerus osteosarcoma remains complicated. The previous study has shown various treatment option with vascularized fibular graft, mega prosthetic replacement, and total elbow arthroplasty has been described in the previous study [4], [5], [6]. In this study, we reported the case of distal humerus osteosarcoma surgically treated with limb salvage surgery by wide excision, cryosurgery followed by total elbow arthroplasty and ORIF with plate and screw as a reconstruction method. We aimed to describe the intraoperative and postoperative outcome of distal humerus osteosarcoma treated with total elbow arthroplasty and ORIF with plate and screw. This case report has been reported in line with the SCARE Criteria [7].

## 2 Case illustration

**Fig. 1:**
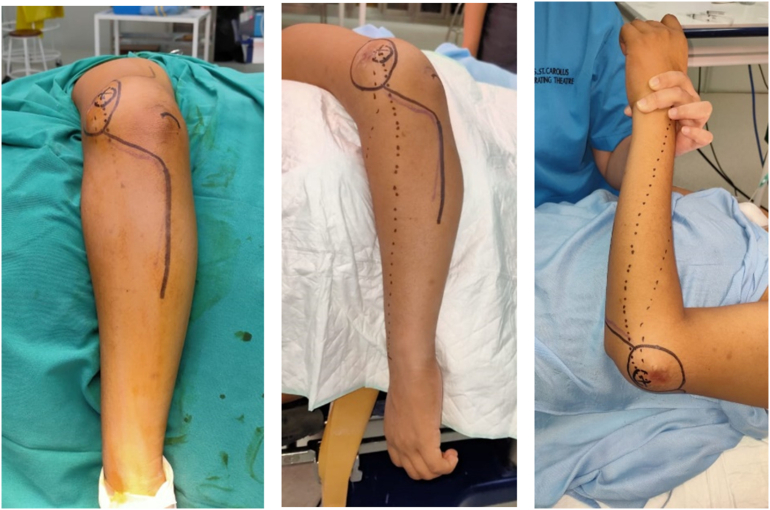
Physical examination showing a mass on the posterolateral aspect of the left elbow.

**Fig. 2:**
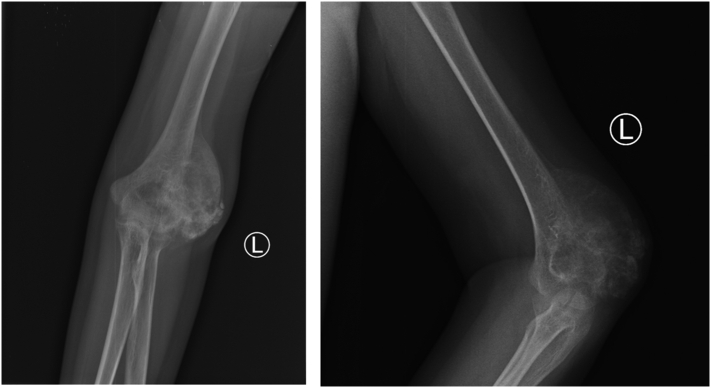
X-ray examination of left elbow showing osteolytic lesion and periosteal reaction.

**Fig. 3:**
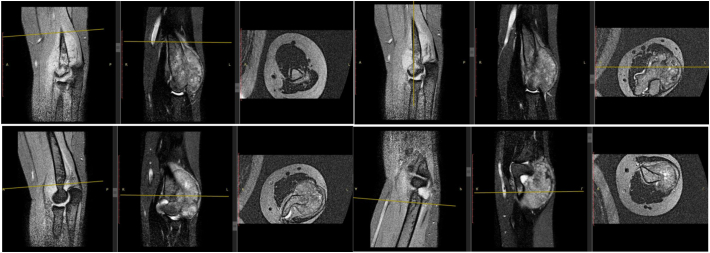
MRI examination of the left elbow showing low intensity signalling on T2 weighted.

**Fig. 4:**
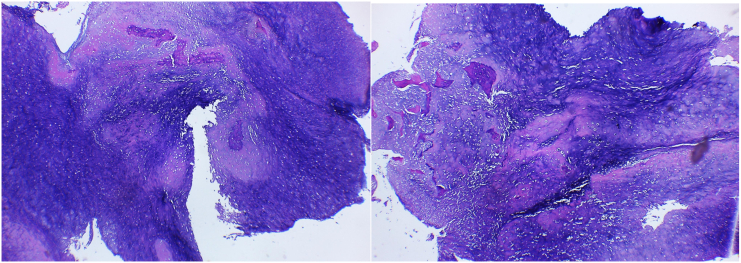
Histopathology examination of the bone tumor showing osteoid matrix.

A 17-year-old boy came with a chief complaint of lump and pain on his left elbow. He previously fell from the stairs about 1 m in height with the elbow hitting the ground, but the pain is subsided in two days. One month later, the patient felt there was a lump with a size of a marbleon the left elbow, which getting bigger to a size of a tenis ball, and was accompanied by pain. General examination showed there was no abnormality but the patient complained of decrease 5 kg of total bodyweight. The local state of the left elbow shows fixed, solid mass on the posterior aspect of the elbow, with the size 14x12cm, 30 cm circumferentially (contralateral 27 cm) (Fig. 1). The range of motion of the left elbow joint could not be measured because of pain. On radiographs obtained at the first examination, an osteolytic lesion and periosteal reaction of the distal humerus was observed (Fig. 2). On magnetic resonance imaging (MRI), a low intensity was observed on T2-weighted imaging (Fig. 3). Computed tomography of the chest showed that there was no abnormality. The patient underwent histopathological and immunohistochemistry examination, with the result of conventional osteosarcoma (Fig. 4). The diagnosis of osteosarcoma was made.

**Fig. 5:**
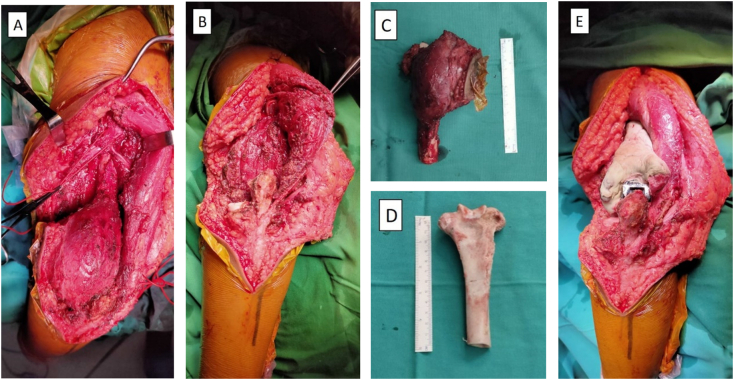
Intraoperative: A) tumor exposed; B) post tumor removal; C) gross pathology; D) bone preparation after cryosurgery; E) final elbow construction.

The patient underwent neoadjuvant chemotherapy with cisplatin, ifosfamide, and adriamycin for 3 cycles. Limb salvage surgery by wide excision, cryosurgery followed by total elbow arthroplasty and ORIF with plate and screw. The patient was positioned right lateral decubitus position under general anaesthesia. The tumor was exposed and shaft humerus osteotomy within 5 cm above the tumor was performed. The radial artery, radial nerve, and ulnar nerve was identified and preserved. The bony segment was resected and prepared for autoclave processing. A thorough debridement of all the tumor and soft tissues from the resected bony segment was performed and the excised bony segment was frozen in pot containing liquid nitrogen for 20 min, thawed at room temperature for 15 min, thawed at room temperature for 15 min. The excised segment then thawed in distilled water for 10 min and reimplanted and fixed in place. Total elbow arthroplasty and ORIF with plate and screw was performed, the bone cement was also added. The implant was secured using advancement of extensor carpi ulnaris muscle and the wound was closed using primary suture.(Fig. 5).

**Fig. 6:**
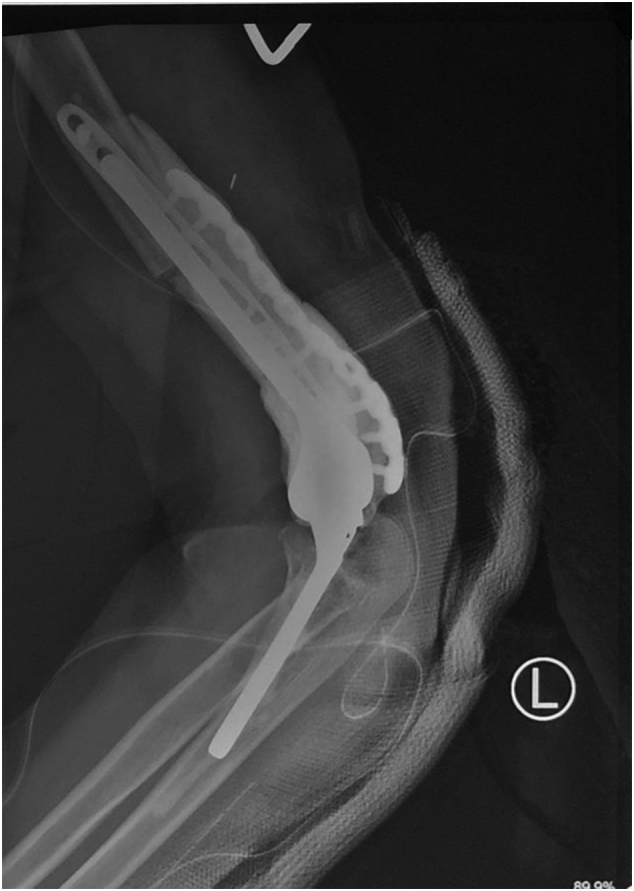
Postoperative X-ray of left humerus.

Postoperatively we performed an X-Ray to evaluate the reconstruction. From the X-ray, the plate and screws are well-fixated (Fig. 6). One month postoperatively, the implant was well-fixated and the patient can slowly move his elbow without limitation.

## 3 Discussion

Although osteosarcoma is the most common primary malignant bone tumor of the long bones, the distal humerus is an uncommon site for osteosarcoma, which could lead to delayed diagnosis and treatment [3], [8]. In this case, the 17-year-old male presents with a painful lump on the left elbow with the history of previous trauma and decrease bodyweight, suggesting malignancy. Series of radiological examination followed with histopathological and immunochemistry examination concluded the diagnosis of conventional osteosarcoma. The previous study has stated the importance of a multidisciplinary approach to diagnose and treat osteosarcoma, especially in an atypical location, as shown in this case [3], [9].

This patient underwent neoadjuvant chemotherapy using adriamycin, cisplatin, and ifosfamide. Neoadjuvant chemotherapy combined with surgery is now the standard treatment for osteosarcoma. It is due to chemotherapy can improve the survival rate of patient with malignant bone tumor [10].

Currently, limb salvage surgery is the standard procedure for treatment of malignant bone tumors. Limb salvage can result in survival rates and disease-free periods that equal those achieved with amputation. Thus, we decide to perform limb salvage surgery in this patient by performing wide excision, cryosurgery and ORIF reconstruction using plate and screw. The use of cryosurgery to preserve the distal segment of the humerus is due to its advantages. Rahman et al. stated that the advantages of reconstruction using tumor-bearing massive frozen autograft treated by liquid nitrogen are its simplicity, osteoconduction, a short treatment time, a perfect fit, easy attachment of tendons and ligaments and desirable bone stock [11]. Several studies with several treatment options have described distal humerus resection and reconstruction. A study by Graci et al. reported en bloc resection followed by intraoperative extracorporeal irradiation and reimplantation with additional nonvascularized fibular graft inside the irradiated bone with the preservation of the joint [12]. Another study by Kamal et al. described an excellent one-year outcome with the reconstruction of the distal humerus using a vascularized fibular graft [5].

This study conducted total elbow arthroplasty and ORIF with plate and screw as a reconstruction modality for distal humerus osteosarcoma. The involvement of elbow joint in this study limits the choice of elbow joint preservation in this study. One-month follow-up has shown excellent functional outcome with complete elbow movement. The use of total elbow arthroplasty in distal humerus tumors has been described in previous studies. Wittig et al. described distal humerus and proximal ulnar reconstruction with modular segmental distal humerus tumor prosthesis [13]. To reconstruct the elbow joint, the distal humeral component comprises a semi-constrained hinge component connected to an ulnar component [13]. The outcome of total elbow arthroplasty was described in his study with local recurrence less than 5%, stable elbow and pain-free, and no case of prosthetic loosening in small-sized case series [13]. Another study by Casadei et al. reported the use of total elbow arthroplasty in 47 subjects with primary or metastatic tumors [14]. He described complications of nerve damage (25%), local recurrence (15%), prosthetic loosening (8%), and infection (2,5%) in patients with total elbow arthroplasty. However, the functional outcome showed excellent results with a mean elbow ROM of 70 in patients with primary tumor and mean MSTS score of 22/30 [14]. In this study, we did not found any early or late complication following total elbow arthroplasty and ORIF with plate and screw.

## 4 Conclusion

Total elbow arthroplasty and ORIF with plate and screw is a favorable reconstruction option for distal humerus osteosarcoma with excellent postoperative functional outcomes.

## Ethical approval

Ethical approval was not required in the treatment of the patient in this report.

## Consent for publication

Written informed consent was obtained from the patient and his parents for publication of this case report and accompanying images. A copy of the written consent is available for review by the Editor-in-Chief of this journal on request.

## Funding

This research did not receive any specific grant from funding agencies in the public, commercial, or not-for-profit sectors.

## Provenance and peer review

Not commissioned, externally peer-reviewed.

## Registration of research studies

Does not need any registration.

## Guarantor

Sigit Daru Cahyadi, MD.

## CRediT authorship contribution statement

Sigit Daru Cahyadi contributes in the study concept or design, data collection, analysis and interpretation, oversight and leadership responsibility for the research activity planning and execution, including mentorship external to the core team.

Uno Surgery Erwin contributes to the study concept or design, data collection and writing the paper.

## Declaration of competing interest

The authors declare no conflicts of interest.
